# Isoflavones: Anti-Inflammatory Benefit and Possible Caveats

**DOI:** 10.3390/nu8060361

**Published:** 2016-06-10

**Authors:** Jie Yu, Xiaojuan Bi, Bing Yu, Daiwen Chen

**Affiliations:** Animal Nutrition Institute, Sichuan Agricultural University, Chengdu 611130, China; yujie@sicau.edu.cn (J.Y.); zsjg214@163.com (X.B.); ybingtian@163.com (B.Y.)

**Keywords:** isoflavones, anti-inflammation, health risks, flavonoids

## Abstract

Inflammation, a biological response of body tissues to harmful stimuli, is also known to be involved in a host of diseases, such as obesity, atherosclerosis, rheumatoid arthritis, and even cancer. Isoflavones are a class of flavonoids that exhibit antioxidant, anticancer, antimicrobial, and anti-inflammatory properties. Increasing evidence has highlighted the potential for isoflavones to prevent the chronic diseases in which inflammation plays a key role, though the underlying mechanisms remain unclear. Recently, some studies have raised concerns about isoflavones induced negative effects like carcinogenesis, thymic involution, and immunosuppression. Therefore, this review aims to summarize the anti-inflammatory effects of isoflavones, unravel the underlying mechanisms, and present the potential health risks.

## 1. Introduction

Inflammation is a biological response stimulated by pathogens, damaged cells, or irritants. Basically, inflammation is an orchestrated protective process to resolve infection, clear out necrotic cells, and trigger tissue repair [1]. However, this process must be rapid, destructive, specific, and well-controlled in order to avoid the over activation of innate immune response, chronic infectious, and/or inflammatory disorders which may cause serious tissue damage [2]. Solid evidence has shown that the development of various diseases including obesity, diabetes, metabolic syndrome, atherosclerosis, rheumatoid arthritis, and cancer are strongly correlated with inflammation [3,4,5,6,7].

Though steroidal or non-steroidal anti-inflammatory drugs are currently used to treat inflammation, these drugs are usually accompanied with unexpected side effects, and also, they are not considered as a good clinical choice for chronic inflammatory disorders [8]. As such, it is urgent to find an effective and safe anti-inflammatory therapy. Numerous epidemiological studies have indicated that vegetarian diets based on fruit and vegetable consumption are beneficial for human health [9,10,11], but the involved food components and the underlying mechanisms still remain unclear. Chinese traditional medicine has had a long history of applying crude plant extracts to treat diseases and disorders exemplified by acute and chronic inflammation [12,13,14]. Recent investigations have demonstrated that the polyphenols from plant sources, in particular flavonoids, exhibit anti-inflammatory activity both *in vitro* and *in vivo* [14,15,16,17]. This not only provides an explanation for the health benefit of vegetarian diets and Chinese medicine, but also identifies potential agents for treating inflammatory disorders, including possible therapy for life-threatening diseases.

**Figure 1:**
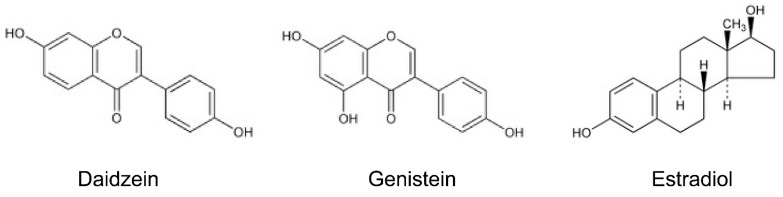
Structure of isoflavones and estradiol.

Flavonoids, which include over 6000 identified family members, are a group of phenolic compounds that are widely distributed in plants and fungus. They have been well-known for their antioxidant, antimicrobial, and anti-inflammatory health benefits [2,18,19]. Within this family, the focus of research has been on a subclass, isoflavones, which are mainly found in soy beans, soy foods, and legumes [20,21,22,23,24] (Table 1). It is well-known that isoflavones act as phytoestrogens to exert pseudohormonal activity by binding to estrogen receptors (ER) in mammals [25,26], and also possess antioxidant, anticancer, antimicrobial, and anti-inflammatory activities just like other flavonoids [27,28,29,30]. Daidzein and genistein are the most common isoflavones, whose characteristic chemical structure (B-ring is linked to the C3 position of the C-ring instead of the C2 position) resembles the structure of estrogens, in particular 17-β estradiol [21] (Figure 1). As such, isoflavones elicit either a weak estrogenic (agonistic) or anti-estrogenic (antagonistic) effect, depending on the levels of endogenous estrogens and ER [31]. Isoflavones block the binding of more potent estrogens, potentially playing a role in preventing hormone-related cancer like breast cancer, cervical cancer, and the male prostate or testicular cancer [32]. Interestingly, the incidence of breast and prostate cancers is lower among Asians in comparison to people in the Western world [33], and this could be related to the significantly different consumption of isoflavones in Asian diets (15–47 mg/day) compared with Western diets (0.15–1.7 mg/day) [34,35].

The physiological effects of flavonoids depend on their bioavailability. Isoflavones are the most absorbable and bioavailable flavonoids, and the bioavailability of genistein is greater than that of daidzein [36,37]. After ingestion, isoflavone glucosides are hydrolyzed to aglycones by glucosidases in the small intestine, where the metabolites are absorbed completely in part or further metabolized into other metabolites, such as equol and *O*-desmethylangolensin, by intestinal microflora in the large intestine [34,38,39]. Next, the metabolites enter the portal vein and undergo further metabolism in the liver [40]. Isoflavones persist in plasma for about 24 h and the average half-life is 6–8 h, as previously reported [41].

Recently, increasing reports about the inhibition of inflammation by isoflavone are available. Meanwhile, some studies have raised concerns that isoflavones induced negative effects like carcinogenesis, thymus atrophy, and immune system abnormalities. Therefore, in this work, we reviewed the anti-inflammatory effects of isoflavones, unraveled the underlying mechanisms, and presented the potential health risks. This document provides some supporting knowledge of fruit and vegetable consumption, and encourages the application of natural medicine.

## 2. Isoflavones and Anti-Inflammatory Effects

In recent decades, extensive epidemiological studies, together with *in vivo* and *in vitro* experiments, have indicated that isoflavones are beneficial for patients with cardiovascular diseases, cancer, osteoporosis, and/or postmenopausal [27,42,43]. Isoflavones were originally speculated to act as an anti-inflammatory agent because genistein down-regulates cytokine-induced signal transduction events in the cells of the immune system [44]. Thereafter, an increasing number of investigations have consistently proven that isoflavones exhibit anti-inflammatory functions.

In mouse models, isoflavone genistein exerts anti-inflammatory properties affecting granulocytes, monocytes, and lymphocytes [44]. Isoflavone-containing diets prevent the inflammation-associated induction of metallothionein in the intestine, and the induction of manganese superoxide dismutase (Mn-SOD) in the liver of mice injected with endotoxin lipopolysaccaride (LPS), and suppresses the intestinal response to inflammation by modulating the action of pro-inflammatory cytokine interleukin (IL)-6 [45]. Kao *et al.* processed soybean cake, a byproduct of the soybean oil industry, into powder in their experiment [46]. They found that the isoflavone powders and genistein standard effectively inhibit LPS-induced inflammation, reduce leukocyte numbers in mouse blood, and lower the production of IL-1β, IL-6, nitric oxide (NO), and prostaglandin E 2 (PGE_2_) in both peritoneal exudates cell supernatant and peritoneal exudates fluid [46]. Soybean methanolic fraction containing isoflavones possess anti-inflammatory activity in the experimental inflammation model of croton oil-induced ear oedema [47]. The unique isoflavone, puerarin can protect the brain from ischemic damage after middle cerebral artery occlusion in rats, and this effect is attributable to the anti-inflammatory properties of puerarin by inhibiting cyclooxygenase-2 (COX-2) expression in astrocyte and microglia [48]. Recently, genistein pre-treatment has been shown to reduce NO and PGE_2_, and suppress the production of d-galactosamine-induced proinflammatory cytokines, including tumor necrosis factor-α (TNF-α) and IL-1β in male Wistar rats [49]. In another study using a guinea pig model of asthma, genistein has been shown to significantly inhibit ovalbumin-induced acute bronchoconstriction, reduce ovalbumin-induced pulmonary eosinophilia and eosinophil peroxidase activity, and attenuate ovalbumin-induced airway hyperresponsiveness to inhaled methacholine [50]. These *in vivo* findings indicate that various isoflavones consistently show anti-inflammatory effects in multiple animal models, while *in vitro* studies using different cultured cells have also demonstrated the anti-inflammatory activity of isoflavones.

In primary cultures of human chondrocytes, genistein pretreatment reduces the LPS-stimulated COX-2 protein level and NO in supernatant without affecting COX-1 protein levels [51]. The genistein suppression of COX-2, but not COX-1, is advantageous since suppressing COX-2 can reduce the production of proinflammatory molecules [51]. Isoflavone daidzein prevents TNF-α-induced increases in pro-inflammatory chemokine Cxcl2 expression and activity, and markedly inhibits TNF-α-induced protein poly-adenosine diphosphate-ribosylation in murine lung epithelial cells [52]. In human monocyte THP-1 cells, daidzein suppresses the LPS-induced IL-6, IL-12, and TNF-α expression levels [53]. Most recently, an investigation reported that genistein prevents homocysteine-induced vascular endothelial cell death, the changes of cell morphology, and the production of reactive oxygen species (ROS), thereby suggesting endothelial cell inflammatory injury is blocked by genistein [54].

In human trials, eight weeks of consuming a soy nut diet (340 mg isoflavones/100 g soy nut) has been shown to reduce the markers of inflammation (e.g., IL-18 and C-reactive protein) and increase plasma nitric oxide levels in postmenopausal women with metabolic syndrome [55]. In postmenopausal women with hypertension, dietary soy nuts (25 g soy protein and 101 mg aglycone isoflavones) improve endothelial function and the underlying inflammatory process [56]. Isoflavone-rich soy foods also decrease serum levels of C-reactive protein in end-stage renal failure patients and interferon γ (IFN-γ) concentrations in healthy volunteers [57,58]. A validated food frequency questionnaire suggests that soy food consumption is related to lower circulating levels of IL-6, TNFα, and soluble TNF receptors 1 and 2 in Chinese women [59]. However, another study indicates that soy protein and isoflavone (either alone or together) have no impact on serum lipids or inflammatory markers, and the reason might be that healthy late postmenopausal women lack the ability to produce equol, which is a metabolite of isoflavones and has more effective biological and pharmacological effects than its prototype [60]. Similar results also indicate that cardiovascular risk reduction with soy nuts is not uniform and may be dependent on the ability to produce equol [61].

The anti-inflammatory properties of isoflavones have been addressed in animals, cell cultures, and clinical trials (Table 2). Moreover, a large number of studies have also elucidated the underlying mechanisms. Though the mechanisms explaining the anti-inflammatory effects of isoflavones still remain unclear, several possibilities have been well unraveled.

## 3. Anti-Inflammatory Mechanisms of Isoflavones

### 3.1. Antioxidative Activities

Free radicals and ROS are produced during normal oxygen metabolism or the physiological process stimulated by exogenous factors, like phagocytosis [62,63]. Excess free radicals and ROS may induce detrimental reactions such as peroxidation of membrane lipids, oxidative damage to nucleic acids and carbohydrates, and oxidation of the susceptible groups in proteins [2,64,65]. Typically, ROS induces the release of various inflammatory mediators, some of which attract neutrophils and other inflammatory cells to promote inflammation and tissue damage [2,66]. Isoflavones are known to directly scavenge reactive species generated by human neutrophils [67,68].

Isoflavones are scavengers of a wide range of reactive oxygen, nitrogen, and chlorine species, but they are relatively resistant to oxidation mediated by the potent oxidant peroxynitrite and hypochlorous acid rather than peroxyl radicals [69,70]. Lai *et al.* reported that isoflavones can scavenge peroxynitrite, a potent oxidant formed *in vivo* from the reaction of nitric oxide with superoxide, to prevent the nitration of tyrosine, while genistein and daidzein dose-dependently inhibit peroxynitrite-mediated low-density lipoprotein (LDL) oxidation [71]. Genistein is able to normalize the increased superoxide anion production and nitrotyrosine formation in streptozotocin-induced type 1 diabetic mice [72]. Isoflavones-enriched *Trifolium* extracts reduce the peroxynitrite-mediated modifications of proteins and diminish peroxidation of lipids in blood platelets [73]. Moreover, oral administration of isoflavones and extracts of soy products decrease serum nitrite, nitrate, and nitrotyrosine levels in LPS-challenged rats [74]. These results suggest that isoflavones scavenge increased free radicals produced by activated macrophages during inflammation, thereby preventing NO reactions with free radicals and the subsequent production of peroxynitrite that can directly oxidize LDL and result in irreversible damage to the cell membrane.

Lately, soy isoflavone has been shown to decrease the concentrations of ROS and malondialdehyde, as well as increase the mRNA expressions and activities of superoxide dismutase and glutathione peroxidase in porcine mammary gland cells and human umbilical vein endothelial cells challenged by hydrogen peroxide [75,76]. These interesting findings, along with a previous report [45], indicate that isoflavones are modulators of enzymatic antioxidants (besides ROS scavengers), and that nuclear factor-erythroid 2 (Nrf2)—a key transcription factor that leads to gene transcription of various antioxidant and phase II detoxifying enzymes—is associated with this modulation [77,78].

### 3.2. Pro-Inflammatory Cytokines and Chemokines Production

**Figure 2:**
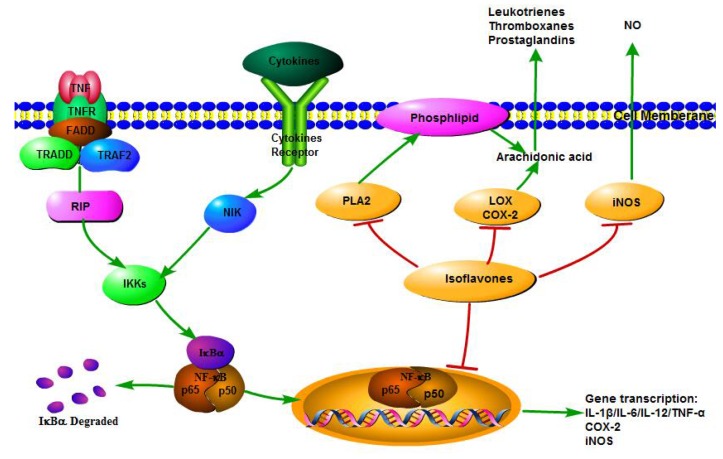
Anti-inflammatory mechanisms of isoflavones. Upon stimulation, cytoplasmic NF-κB is activated by IκB kinase (IKK). Then, free NF-κB translocates into the nucleus and activates the transcription of target genes including pro-inflammatory cytokines and chemokines, inducible nitric oxide synthases (iNOS), and cyclooxygenase 2 (COX-2). Isoflavones decrease the production of these pro-inflammatory contributors by inhibiting the NF-κB transcriptional system. Also, isoflavones modulate arachidonic acid (AA) metabolism and NO production by inhibiting the protein levels and activities of pro-inflammatory enzymes (phospholipase A2 (PLA2), lipoxygenase (LOX), COX-2, and iNOS). The metabolites of AA—including prostaglandins (PG), leukotrienes and thromboxances, and NO—are crucial mediators of inflammation.

Inhibiting the production of pro-inflammatory cytokines and chemokines such as IL-1β, IL-6, IL-12, and TNF-α is a major mechanism by which isoflavones exert anti-inflammatory functions (Figure 2). Macrophages are the major cells releasing pro-inflammatory cytokines. Isoflavone pretreatment has been shown to regulate gene transcription of cytokines and inflammatory markers, and to inhibit the overproduction of NO and PGE_2_ induced by LPS plus IFN-γ in RAW 264.7 macrophages by transcriptomic profiling [79]. A recent study demonstrated that genistein effectively suppresses the LPS-stimulated overproduction of IL-6 and TNF-α in RAW 264.7 macrophages [80]. Irisflorentin, a naturally occurring isoflavone, also significantly inhibits TNF-α, IL-1β, and IL-6 at both transcriptional and translational levels in LPS-induced RAW 264.7 macrophages [81]. Moreover, isoflavones fraction suppresses TNF-α-induced IL-8 secretion, and inhibits the IL-8 promoter activity in human intestinal epithelial Caco-2 cells, thereby suggesting isoflavones are promising food components for preventing intestinal inflammation [82]. Daidzein is found to inhibit IL-6 and IL-8 production in Toll-like receptor (TLR)2, and TLR4-stimulated monocytes in a dose-dependent manner [83]. In addition to *in vitro* evidence, isoflavones suppress pro-inflammatory cytokines and chemokines during inflammatory reaction in diverse animal models and humans [21,45,49,84]. In a murine model of human inflammatory demyelinating disease, genistein impressively down-modulates IFN-γ and IL-12 cytokine in the brain, thereby suggesting genistein might be a potential therapy for multiple sclerosis [85]. Genistein supplementation significantly decreases the mRNA levels of TNF-α and IL-1β, and alleviates hepatic inflammation and fibrosis in nonalcoholic fatty liver disease db/db mice [86]. In a randomized phase II trial, consumption of soy isoflavone enriched bread was shown to reduce pro-inflammatory cytokines with limiting inflammation and results in the suppression of myeloid-derived suppressor cells in men with prostate cancer [84].

### 3.3. Pro-Inflammatory Enzyme Activities

Arachidonic acid (AA) related enzymes such as cyclooxygenase (COX), lipoxygenase (LOX), and phospholipase A2 (PLA2), plus nitric oxide synthases (NOSs) are typical pro-inflammatory enzymes which catalyze the production of vital inflammatory mediators including prostaglandins (PG), leukotrienes, AA, and NO [87,88,89,90].

When a general inflammatory response is induced, cytosolic PLA2 action triggers the release of AA from membrane phospholipids [91]. AA is then metabolized to PG and thromboxances by COX, or leukotrienes by LOX [21]. Isoflavones can modulate AA metabolism by inhibiting the protein levels and activities of AA related enzymes, providing a possible mechanisms to explain the anti-inflammatory functions of isoflavones (Figure 2).

Genistein inhibits secretory PLA2 activity of both inflammatory exudates and snake venoms in a concentration dependent manner [92]. An isoflavone isolated from *Harpalyce brasiliana* Benth, harpalycin 2, has been found to inhibit the enzymatic, edematogenic, and myotoxic activities of secretory PLA2 from various snake venoms, and harpalycin 2 significantly suppresses PLA2-induced edema at the initial step [93]. Furthermore, docking calculations indicated that the residues His48 and Asp49 powerfully interact with harpalycin 2 through hydrogen bonds in the active site of PLA2 [93].

There are two isoforms of COX in mammals. COX-1 is a constitutive enzyme widely distributed in most cell types to catalyze PG synthesis in normal physiological conditions, whereas COX-2 is an inducible enzyme specifically presented in inflammatory cells to respond to bacterial LPS and/or pro-inflammatory cytokines [21,94]. Genistein and daidzein alleviate aortic endothelium-dependent contraction to acetylcholine in spontaneously hypertensive rats by inhibiting COX activity and reducing the endothelial prostaglandin H2 release [95]. Isoflavones from soybean and tempeh ameliorate scopolamine-induced amnesia in rats by disrupting COX-2 action and preventing neuroinflammation [96]. Other than *in vivo* inhibitory effects of isoflavones on COX-2, *in vitro* experiments have also demonstrated the suppressive effect of isoflavones on COX-2 expression and activity. Genistein and daidzein inhibit phorbol 12-myristate 13-acetate (PMA) induced COX-2 transcription and protein expression in the breast cell line MCF-7 culture system [97]. Nonetheless, isoflavones suppression of the COX-2 promoter transactivation may be dependent on AP-1/CREB binding [97]. In the human gastric cancer cell line BGC-823, genistein treatment markedly decreases COX-2 protein levels in a dose-dependent manner, inhibiting cell proliferation and inducing apoptosis [98].

LOX metabolizes AA to leukotrienes and hydroxyl acids, and is involved in the onset and the development of a diverse number of human diseases [21]. The inhibitory effect of isoflavones has been identified. Genistein and daidzein are noncompetitive inhibitors of the activity of 5-LOX from human polymorph nuclear leukocytes in a concentration dependent manner [99]. Enzymatic evaluation indicates that isoflavone texasin is an effective 5-LOX inhibitor [100]. An *in vitro* experiment has shown that isoflavones suppress the activities of human platelet 12-LOX, reticulocyte 15-LOX-1, and epithelial 15-LOX-2, and a catechol group in ring A of isoflavones might be critical for the inhibitory effect according to a docking study [101]. Mechanically, isoflavones are able to convert the active form (ferric state) of LOX to the resting form (ferrous state) or prevent the activation of the resting state, thereby regulating LOX activity [2].

The NOS family includes endothelial NOS (eNOS), neuronal NOS (nNOS), and inducible NOS (iNOS), producing NO, a ubiquitous cellular mediator of physiological and pathological processes, from l-arginine. Other than constitutively producing low physiological levels of NO by eNOS and nNOS, iNOS is induced by bacterial LPS and/or pro-inflammatory cytokines to overproduce NO in macrophages and other cells [102,103]. Isoflavones have been shown to inhibit NO production by iNOS from certain cells without affecting eNOS and nNOS, making isoflavones the desirable anti-inflammatory agents. Primary isoflavones genistein, daidzein, and glycitein dose-dependently suppress NO production, and inhibit both the activity and expression of iNOS in LPS-stimulated murine macrophages [104]. In primary cultured microglia and the BV2 microglial cell line, isoflavones repress LPS-induced iNOS expression at both the transcriptional and post-transcriptional level [105,106]. Genistein administration is also found to prevent the increases of iNOS activity in diabetic wound tissues in a dose-dependent manner [72]. On the contrary, a previous study showed that genistein and daidzein activate iNOS and enhance NO production through the estrogen receptor (ER) pathway in RAW 264.7 macrophages [107]. In light of these experimental data, isoflavones may act to maintain the production of NO in normal physiological conditions, but prevent the overproduction of NO through inhibiting the expression and activity of iNOS in a pathological state.

### 3.4. NF-κB Transcriptional System

A number of cellular signaling cascades are involved in the anti-inflammatory activity of isoflavones. The isoflavones activation of adenosine monophosphate-activated protein kinase (AMPK), protein kinase C (PKC), and mitogen-activated protein kinase (MAPK) regulate the DNA-binding capacity of transcription factors like NF-κB and activator protein-1 (AP-1) and lead to the subsequent increases of target gene transcription [78,80,105,106,108]. In addition, NF-κB is the major effector in inflammatory and immune response [109]. Thus, the NF-κB transcriptional system is considered to be the control point in the anti-inflammatory function of isoflavones (Figure 2).

Upon stimulation, the inhibitor of NF-κB, IκB, is phosphorylated and then degraded to set NF-κB free. Cytoplasmic NF-κB subsequently translocates into the nucleus and activates the transcription of target genes including pro-inflammatory cytokines and chemokines, and adhesion molecules, iNOS, and COX-2 [110]. Furthermore, the Ser536 on the p65 subunit of NF-κB can be phosphorylated and activated by IκB kinase (IKK) after LPS stimulation [111]. This IκB independent pathway is also a mechanism by which NF-κB regulates gene transcription and pro-inflammatory cytokines production in addition to the canonical regulation of NF-κB induction [112]. The isoflavone genistein, but not daidzein, represses TNF-α-induced NF-κB activation in human peripheral blood lymphocytes, and an isoflavone mixture also inhibits TNF-α-induced NF-κB activation and oxidative DNA damage in healthy men [113]. However, another study demonstrates that daidzein attenuates ischemia/reperfusion-induced myocardial damage via inhibiting NF-κB activation in rats [114]. A previous study indicated that genistein attenuates hemolysate-induced NF-κB p65 translocation in rat brain microvascular endothelial cells, which in turn suppresses the expression levels of pro-inflammatory and adhesion molecules [115]. Recently, genistein supplementation was shown to accelerate diabetic wound closure rate and decrease pro-inflammatory cytokines, iNOS, and COX-2 by inhibition of IκB phosphorylation, and subsequent NF-κB activation [116], thereby suggesting isoflavones regulate NF-κB activation through both IκB dependent and independent pathways.

## 4. Potential Health Risks of Isoflavones Intake

Numerous studies have provided a wealth of information on the anti-inflammatory effects and the underlying mechanisms of isoflavones, however, some studies have raised concerns about isoflavones induced negative effects.

As mentioned above, isoflavones are usually classified as phytoestrogens which have both estrogenic and anti-estrogenic effects. It has been suggested that isoflavones can either activate ERα to promote cell proliferation or bind to ERβ to promote apoptosis [117]. So even if isoflavones are widely known as prospective compounds for preventing carcinogenesis [27,118], especially breast cancer [27,119,120,121,122], they have been shown to promote tumor development and elevate cancer risk in some research studies [123]. Previous studies discovered that genistein increases the percentage of proliferative cells in tumors, enhances tumor multiplicity, and elevates the weight of estrogen-dependent mammary adenocarcinomas in rats [124,125]. Moreover, neonatal exposure to genistein increases uterine fibroid incidence and multiplicity in Eker rats via impairing myometrial DNA repair capacity [126,127]. Administration of genistein or daidzein also induces apoptosis in human prostate hyperplasia cells, indicating that individual isoflavones may be cytotoxic for non-cancerous cells [128]. There are two hypotheses put forward to explain the seemingly contradictory health effects. One is a balance between two opposing receptors (ERα and ERβ) in cells. The eventual outcome might be an interactive effect among many complex mechanisms depending on the factors which change the ER condition in the cell, including cellular context, balance between ER subtypes, or co-activators and co-repressors, and so on. Another hypothesis is that epigenetics, such as DNA methylation, histone modification, and miRNA expression patterns, could adjust the role of the effect of isoflavones on cancer [129]. Therefore, the relationship between isoflavones and cancer does not always appear to be positive, and the potential carcinogenic risk should be considered.

Similarly, isoflavones offer immunologic benefits to various animals, but a few studies have raised the possibility of immunosuppression by isoflavones. Genistein injection leads to dose-dependent decreases in thymic weight up to 80%, reduces thymocyte numbers, increases thymocyte apoptosis, and suppresses humoral and cellular immune response through either ER- or non-ER-mediated pathways in ovariectomized juvenile mice [130,131]. The most recent research has demonstrated that dietary soy isoflavones during the prenatal period significantly cause lymphocytic depletion in white pulp of the spleen and decrease thymic relative weights in rat offspring [132].

Long-term consumption is one of the key factors of consideration concerning the safety of isoflavones. Epidemiological data from Hawaiian populations indicated that there is an association between long-term soy consumption and Kawasaki disease (KD), and soy isoflavones are involved in KD pathogenesis [133]. In addition, a recent study shows that isoflavone exposure via consumption of soy-based infant formula negatively affects the long-term development of infants [134]. Long-term exposure to genistein promotes the growth of breast cancer cells and results in soy protein isolate-induced non-regressing tumors with more aggressive and advanced growth phenotypes [135].

Another key factor which may elevate the healthy risk of isoflavones is high dosage. A recent meta-analysis shows that high intake of soy isoflavones increases the risk of cancer recurrence in epidermal growth factor receptor-2 (HER-2)-positive breast cancer in Korean women [136]. Supplementation with a high dose (150 mg/BW) of genistein during a 21-day gestation period induces adverse effects on the reproductive organs of first generation (F1) murine weanling-stage offspring [137]. Another dose range-finding study was conducted as a prelude to a multi-generation bioassay to assess potential toxicities associated with genistein consumption [138]. It has been demonstrated that exposure to over 25 mg/kg feed of genistein significantly inhibits breast tissue hyperplasia and hypertrophy, decreases offspring birth weight, reduces male offspring prostate weight, delays testicular developmental, and induces sperm retardation and deformation [138]. Studies by our group indicated that the biological activity of daidzein in pigs depends on dosage [139]. Dietary supplementation with a higher dose of daidzein (400 mg/kg feed) decreases body weight gain and increases the splenic damage index in weaned pigs [139]. *In vitro* studies also show contrasting effects, that isoflavones show a strong cardioprotective activity at low concentrations and cardiotoxic activity at high concentrations [140]. Furthermore, high concentrations (such as 200 μM) of genistein exert pro-oxidant potential in primary porcine muscle cells by enhancing ROS production in a 5-LOX-dependent manner [141].

In summary, the potential risks of isoflavones have been highlighted in some investigations. However, the current literature is quite incomplete and sometimes contradictory. The negative effects of isoflavones may rely on diverse factors such as age at the time of exposure and the hormonal environment.

## 5. Conclusions

As shown by this summary of abundant evidence, isoflavones exhibit impressive anti-inflammatory properties in various animal models, and even in humans, through increased antioxidative activities, NF-κB regulation, and reduced pro-inflammatory enzymes activities and cytokine levels, thereby encouraging the application of isoflavones in a range of inflammatory diseases. Noticeably, the potential carcinogenic and immunosuppressive effects of isoflavone raise concerns about the risks of isoflavone consumption. However, extensive evaluations are still warranted to explore the exact mechanisms and answer the safety question. At the present time, this concise work may renew certain knowledge of isoflavones in inflammatory process, and may draw the attention of the public to vegetarian diets and natural medicine.

## Figures and Tables

**Table 1:** Approximate content of isoflavones in main sources.

Sources	Approximate Contents (mg/100 g)	Reference
Soy bean	26–381	[20,22]
Roasted soy bean	246	[23]
Soy tempe	148	[23]
Soy flour	83–466	[22,23]
Tofu	8–67	[20,22]
Miso	25–89	[22]
Tempeh	86.5	[20]

**Table 2:** *In vivo* and *in vitro* anti-inflammatory effects of isoflavones.

Treatments	Dosage	Effects		Models	Reference
Genistein	30 mg/kg every 2nd day	Granulocytes, monocytes, and lymphocytes	↓	Mice	[44]
Soybean cake	0.3 mL aqueous solution	leukocyte number, IL-1β, IL-6, NO, and PGE_2_	↓	Mice	[46]
Soybean methanolic fraction	2.5 mg/kg	Acute toxicity Inflammation	↓	Mice	[47]
Puerarin	12.5 mg/kg	COX-2, astrocyte and microglia	↓	Mice	[48]
Genestein	5 mg/kg·BW/day	iNOS, COX-2, NF-ĸB, IKK α/β, MAPK	↓	Rats	[49]
Genestein	15 mg/kg	Bronchoconstriction, peroxidase	↓	Guinea pigs	[50]
Daidzein	400 mg/kg	Cxcl2 Poly-adenosine Diphosphate-ribosylation	↓	Murine lung epithelial cells	[52]
Genistein	10, 50, 100 μM	Cell morphology, ROS	↓	Vascular endothelial cells	[54]
Genistein	894 mg/kg	Metallothionein, IL-6, STAT3 Pro-inflammatory cytokine	↓	Mice, Caco-2 cells	[45]
		Mn-SOD	↑		
Green soybean extract	50 mg/mL	IL-6, IL-12 and TNF-α expression levels	↓	Human THP-1	[53]
